# Synthesis of Y_2_O_3_ Oxide Dispersion-Strengthened Ti-6Al-2Sn-4Zr-2Mo Alloy Powder by In Situ Gas Atomization Method

**DOI:** 10.3390/ma18030521

**Published:** 2025-01-23

**Authors:** Hyeon-Tae Im, Ryun-Ho Kwak, Sung-Min Park, Chang-Soo Park, Hyung-Ki Park

**Affiliations:** Functional Materials and Components R&D Group, Korea Institute of Industrial Technology, Gangneung 25440, Republic of Korea; iht89@kitech.re.kr (H.-T.I.); kwag01@kitech.re.kr (R.-H.K.); park3875@kitech.re.kr (S.-M.P.); zionzia@kitech.re.kr (C.-S.P.)

**Keywords:** in situ gas atomization, oxide dispersion-strengthened alloy, Ti-6Al-2Sn-4Zr-2Mo alloy, Y_2_O_3_ oxide particles, additive manufacturing

## Abstract

Oxide dispersion-strengthened (ODS) alloys demonstrate enhanced mechanical properties at elevated temperatures and show potential as next-generation powder materials for additive manufacturing. These alloys can mitigate defects such as micropores and cracks by regulating solidification and grain growth behaviors during the additive manufacturing process. This study investigates the fabrication technology for ODS Ti-6Al-2Sn-4Zr-2Mo (Ti6242) alloy powder to achieve uniform oxide distribution within the alloy powders. Thermodynamic calculations were employed to determine the optimal Ti6242–Y_2_O_3_ composition for in situ gas atomization, ensuring complete dissolution of the oxide in the Ti6242 molten metal and subsequent reprecipitation upon cooling. A rod-shaped ingot was produced via vacuum arc melting, resulting in coarse Y_2_O_3_ precipitating along the grain boundaries. The powder was fabricated through an electrode induction gas atomization method, and the ODS Ti6242 powder exhibited a spherical shape and a smooth surface. Cross-sectional analysis revealed the uniform distribution of Y_2_O_3_ oxide particles, measuring several tens of nanometers in size, within the alloy powder. This research demonstrates the successful synthesis of oxide-integrated ODS Ti6242 alloy powder through the in situ gas atomization method, potentially advancing the field of additive manufacturing for high-temperature applications.

## 1. Introduction

Titanium (Ti) alloys are widely utilized in chemical plants and as biomaterials due to their high specific strength, corrosion resistance, and biocompatibility [1,2,3]. Among these, the α + β dual-phase Ti-6Al-4V (Ti64) alloy is extensively applied in various mechanical components owing to its high mechanical properties at room temperature [4,5]. However, the Ti64 alloy exhibits a rapid decrease in mechanical properties as temperature increases, limiting its maximum service temperature to approximately 350 °C [6].

Ti-6Al-2Sn-4Zr-2Mo (Ti6242), a near–α Ti alloy, displays high mechanical properties attributed to substitutional solid solution strengthening. Ti6242 exhibits greater high-temperature strength than Ti64 and demonstrates high creep resistance due to its low self-diffusion coefficient and a limited number of dislocation slip systems [7]. With a maximum service temperature of 538 °C, Ti6242 is suitable for high-temperature applications such as aircraft gas turbines and power generation [8]. However, despite its greater high-temperature mechanical properties than other Ti alloys, Ti6242’s applications remain limited due to its lower maximum service temperature and high-temperature mechanical properties relative to Ni-based superalloys.

Low thermal conductivity and high oxidation reactivity complicate parts manufacturing using conventional machining and cutting methods [9,10]. Recent studies on additive manufacturing of Ti alloys have been actively conducted to overcome these limitations [11,12,13]. Additive manufacturing technology offers the advantages of manufacturing parts with complex internal structures and near-net shapes [14,15]. However, the repeated melting of metal powders in additive manufacturing can lead to defects such as micropores and cracks within the part, resulting in deteriorated mechanical properties [16,17,18]. These defects significantly diminish toughness and fatigue properties, thereby decreasing component reliability.

Martin et al. [19] reported overcoming these defects in additive manufacturing by utilizing metal powders combined with dispersion materials. Dispersion nanoparticles were attached to the surface of Al7075 alloy powder using an electrostatic method. The nanoparticles controlled solidification and grain growth behavior during the additive manufacturing process, greatly reducing pores and cracks. This reduction in defects increased elongation, while forming a fine isotropic microstructure and nanoparticle dispersion greatly enhanced mechanical strength.

Smith et al. [20] investigated the additive manufacturing of oxide dispersion-strengthened (ODS) alloy, dispersing yttria (Y_2_O_3_) in NiCoCr high-entropy alloy. Y_2_O_3_ nanoparticles were attached to the NiCoCr alloy powder using a mixer. The oxide dispersion significantly improved both the room temperature and high-temperature strength of additive manufactured samples. The tensile strength of NiCoCr ally at 1093 °C increased from 65 MPa to 90 MPa with the dispersion of 1 wt% Y_2_O_3_.

These studies demonstrate the potential of dispersion materials to control defects and improve physical properties in additive manufacturing. However, previous studies fabricated dispersion-strengthened powders by attaching dispersion particles to the surface of metal alloy powders using wet chemical or mixing methods, which resulted in uneven distribution of dispersion particles. In addition, these methods presented challenges in powder reusability due to dispersion materials falling off during powder flow.

Im et al. [11] reported on the fabrication of ODS Ti powder with uniformly dispersed nanosized oxide particles inside spherical pure Ti powder to overcome this limitation. By examining thermodynamic oxidation and reduction reactions, a Ti–Y_2_O_3_ composition was derived in which oxide dissociated into the Ti molten metal above the melting point of Ti and reprecipitated within the Ti upon solidification. Using this reaction, an in situ gas atomization method was developed in which oxide decomposes in molten metal during melting and then uniformly precipitates as nanoparticles within the powder through rapid cooling during the gas atomization process.

Ti6242 alloy, a high-temperature Ti material, has the potential for broader application in high-temperature components if its high-temperature properties can be enhanced through oxide dispersion. Moreover, the use of metal powders with oxide particles may reduce defects, such as micropores and cracks, in additive manufactured parts. Therefore, this study focuses on the fabrication of ODS Ti6242 powder, in which Y_2_O_3_ nanoparticles were uniformly dispersed within Ti6242 powder using the in situ gas atomization method. To prepare oxide-integrated Ti6242 powder, thermodynamic calculations were conducted on the oxidation and reduction reactions involving the Ti6242 matrix and Y_2_O_3_. The ODS Ti6242 powder was fabricated by an electrode induction gas atomization (EIGA) method, and the formation of Y_2_O_3_ particles inside the Ti6242 powder was observed through microstructure analyses.

## 2. Materials and Methods

The fabrication of powder using EIGA initially required the preparation of a Ti6242–Y_2_O_3_ alloy electrode using vacuum arc remelting (VAR) (Samhan, Paju, Republic of Korea) equipment. Raw materials for the melting process consisted of Ti6242 master alloy with a 0.1% oxygen concentration and a 99.99% purity Y_2_O_3_ powder with an average particle size of 4.5 μm. The oxygen concentration of the Ti6242 mater alloy was analyzed by an inert gas fusion infrared absorption method using an oxygen analyzer (736 series, LECO, St. Joseph, MI, USA). The oxygen analyzer was calibrated using a titanium reference sample prior to the analysis. A rod ingot for EIGA was prepared by adding 0.7 wt% Y_2_O_3_ to the Ti6242 alloy, and the VAR chamber was evacuated to a high vacuum of 10^−5^ torr and subsequently filled with high-purity argon (Ar) gas to facilitate melting and casting. The resulting alloy, referred to as Ti6242–0.7Y_2_O_3_, was fabricated into a rod-shaped ingot with a diameter of 30 mm and a height of 200 mm using a water-cooled copper crucible.

ODS Ti6242 powders were fabricated via EIGA using the Ti6242–0.7Y_2_O_3_ rod ingot. The diameter of the rod ingot was 30 mm. The EIGA chamber was evacuated to a high vacuum of 10^−5^ torr by vacuum pumps and filled with a high-purity Ar gas. The rod ingot was rotated at 6 rpm for uniform melting, and its descending speed was 40 mm/min. Considering the diameter and descending speed of the rod, 125 g of ingot was melted and fabricated into powders per min. The rod ingot was melted using an induction power of 70 kW, and a high-purity Ar gas at 50 bar was employed to atomize the liquid metal stream into powder. Subsequently, the powder was classified into particle sizes ranging from 53 to 150 µm using a sieve.

Thermodynamic reactions of Ti and oxides were calculated using the Thermo-Calc 2021b software. The oxidation driving force of the reaction in which oxygen dissolves in the pure Ti and Ti6242 alloy matrix was derived using the TCTI5 database. The oxygen concentration for the pure Ti and Ti6242 alloy was set to 0.1 wt%, corresponding to the oxygen concentration of the Ti6242 raw material used in the study. The oxidation driving force of the reaction in which a pure metal forms an oxide was derived using the SSUB5 compound database. In addition, the equilibrium phase fraction as a function of temperature was calculated to elucidate the dissolution and precipitation behavior of Y_2_O_3_ in the Ti6242 alloy matrix.

Microstructural analyses of the rod ingot and powder were conducted using a field-emission scanning electron microscope (FE–SEM) (QUANTA FEG 250, FEI, Hillsboro, OR, USA) equipped with energy-dispersive spectroscopy (EDS) (Octane Elite EDS, AMETEK, Berwyn, PA, USA). The phase formation was investigated using an X-ray diffractometer (XRD) (Empyrean, Malvern Panalytical, Malvern, Worcestershire, UK). Oxide particles precipitated within the ODS Ti6242 powder were observed through a field-emission transmission electron microscope (FE–TEM) (Titan G2 ChemiSTEM Cs Probe, FEI, Hillsboro, OR, USA). Samples for TEM analysis were prepared using a focused ion beam (Versa 3D DualBeam, FEI, Hillsboro, OR, USA).

## 3. Results and Discussion

**Figure 1:**
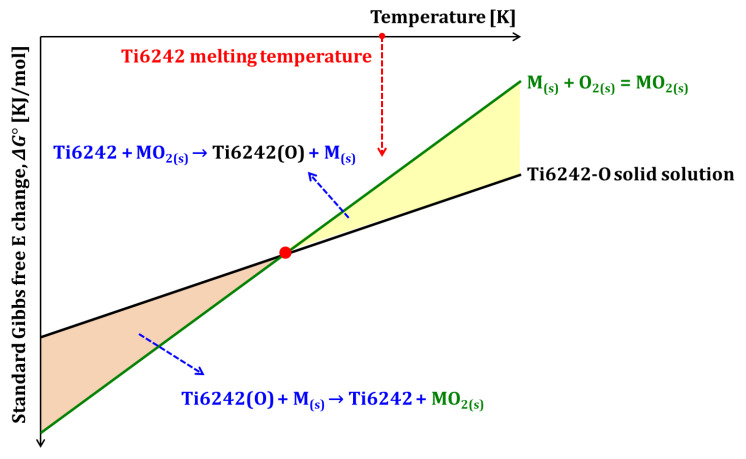
Schematic showing a thermodynamic approach for fabricating the ODS Ti6242 powder by in situ gas atomization method.

Figure 1 presents a schematic diagram illustrating the thermodynamic concept underlying the fabricating of ODS Ti6242 powder with internally distributed oxide via the in situ gas atomization method. The x-axis shows the temperature, while the y-axis shows the negative value of the standard Gibbs free energy change (Δ*G*°) for the oxidation reaction. The black line depicts the driving force for the oxidation reaction in which oxygen is dissolved in the Ti6242 alloy matrix. In contrast, the green line represents the driving force for the oxidation reaction forming metal oxides. In this graph, the y-intercept corresponds to the standard enthalpy change (Δ*H*°), and the slope represents the negative value of the standard entropy change (Δ*S*°). Among the two oxidation reactions, the slope of the reaction involving oxygen dissolution in the Ti6242 alloy matrix represents smaller reactions. This is due to the lower volume change associated with oxygen dissolution compared to oxide formation during the oxidation reaction, resulting in a relatively smaller Δ*S*° [21,22].

The oxidation driving force for metals to form oxides varies depending on the elements. Figure 1 illustrates the relationship where the green and black lines intersect below the melting point. An oxide satisfying this relationship would dissolve in the Ti6242 molten metal above the melting temperature of the Ti6242 and finely reprecipitate in fine particles within the Ti6242 powder during the rapid cooling of the gas atomization process.

**Figure 2:**
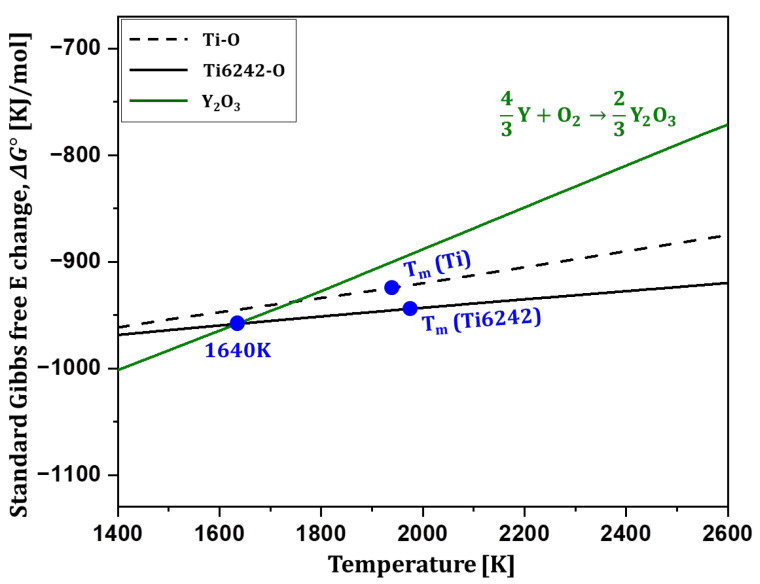
Oxidation driving force for the reaction in which oxygen is dissolved in pure Ti (black dotted line) and Ti6242 (black solid line). Green line represents the oxidation driving force of Y to form Y_2_O_3_ oxide.

To derive a combination of Ti6242 and an oxide that satisfies this relationship, the driving force for the oxidation reaction involving oxygen dissolution in the Ti6242 matrix was calculated and presented in Figure 2. The black dotted and solid lines represent the oxidation driving force for the reaction in which oxygen is dissolved in pure Ti and Ti6242 alloy matrix, respectively. The comparison of Δ*G*° values reveals that the oxidation reaction exhibits a higher driving force when involving oxygen dissolution in the Ti6242 matrix. The Ti6242 alloy, composed of Ti with added aluminum (Al), tin (Sn), zirconium (Zr), and molybdenum (Mo), was subjected to detailed analysis to elucidate the impact of these alloying elements on the oxidation driving force.

**Figure 3:**
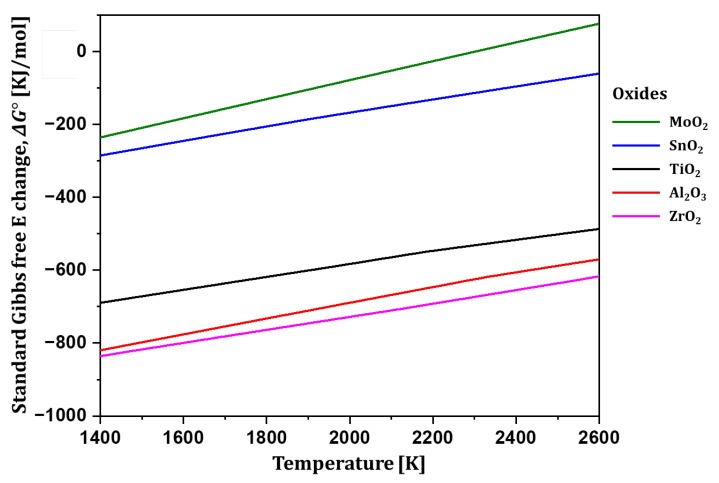
Oxidation driving force for oxide formation of Ti, Al, Sn, Zr, and Mo elements.

Figure 3 shows the oxidation driving force for reactions in which Ti, Al, Sn, Zr, and Mo form oxides. Al and Zr demonstrate a higher oxidation driving force for forming oxides than Ti, while Mo and Sn have a lower oxidation driving force. The Ti6242 alloy contains 6 wt% Al and 4 wt% Zr, elements with higher oxidation driving forces than Ti, along with 2 wt% each of Sn and Mo, which have lower oxidation driving forces than Ti. Consistent with the driving forces for oxide formation, the addition of Al and Zr increases the driving force for oxidation in which oxygen is dissolved in the matrix. Consequently, the incorporation of elements with a higher oxidation driving force than Ti enhances the oxidation driving force for oxygen dissolution in the Ti6242 matrix compared to pure Ti (Figure 2).

In Figure 2, the green solid line represents the oxidation driving force of yttrium (Y) to form Y_2_O_3_. The oxidation driving force for oxygen dissolution in the Ti6242 matrix (black solid line) and the oxidation driving force for Y_2_O_3_ formation (green solid line) intersect at 1640 K. Above 1640 K, the higher oxidation driving force for oxygen dissolution in the Ti6242 matrix promotes Y_2_O_3_ dissolution into the Ti6242 matrix. Conversely, below 1640 K, the higher oxidation driving force for Y to form oxide results in oxygen from the Ti6242 matrix combining with Y to form Y_2_O_3_. This relationship satisfies the thermodynamic conditions for oxide dispersion inside the powder via in situ gas atomization, as presented in Figure 1.

The dissolution behavior of Y_2_O_3_ in the Ti6242 matrix varies depending on the Y_2_O_3_ content. Im et al. [12] reported a study on dispersing Y_2_O_3_ inside Ti64 alloy powders. From the thermodynamic calculation results, it was confirmed that the dissolution temperature of Y_2_O_3_ in the Ti64 matrix increased with higher Y_2_O_3_ content. When the dissolution temperature of Y_2_O_3_ exceeds the solidus temperature at which the liquid phase is formed, oxides remain in the molten metal. In this case, oxides may be lost to slag and aggregate during the EIGA process, causing problems with non-uniform composition. In Ti64 alloy, when the oxygen concentration in the Ti64 matrix was 0.1 wt% and the Y_2_O_3_ content exceeded 1.5 wt%, the dissolution temperature of Y_2_O_3_ became higher than the solidus temperature. Therefore, the Y_2_O_3_ content needs to be controlled below this level. Conversely, if the oxide content in the ODS alloy is too low, the ODS effect may be minimal. Previous studies on ODS alloys in Ni-based superalloys and steels have shown that excellent high-temperature mechanical properties could be achieved when 0.7 wt% oxide was added [23,24,25]. Based on this, thermodynamic analysis and powder fabrication experiments were conducted on the Ti6242 alloy containing 0.7 wt% Y_2_O_3_.

**Figure 4:**
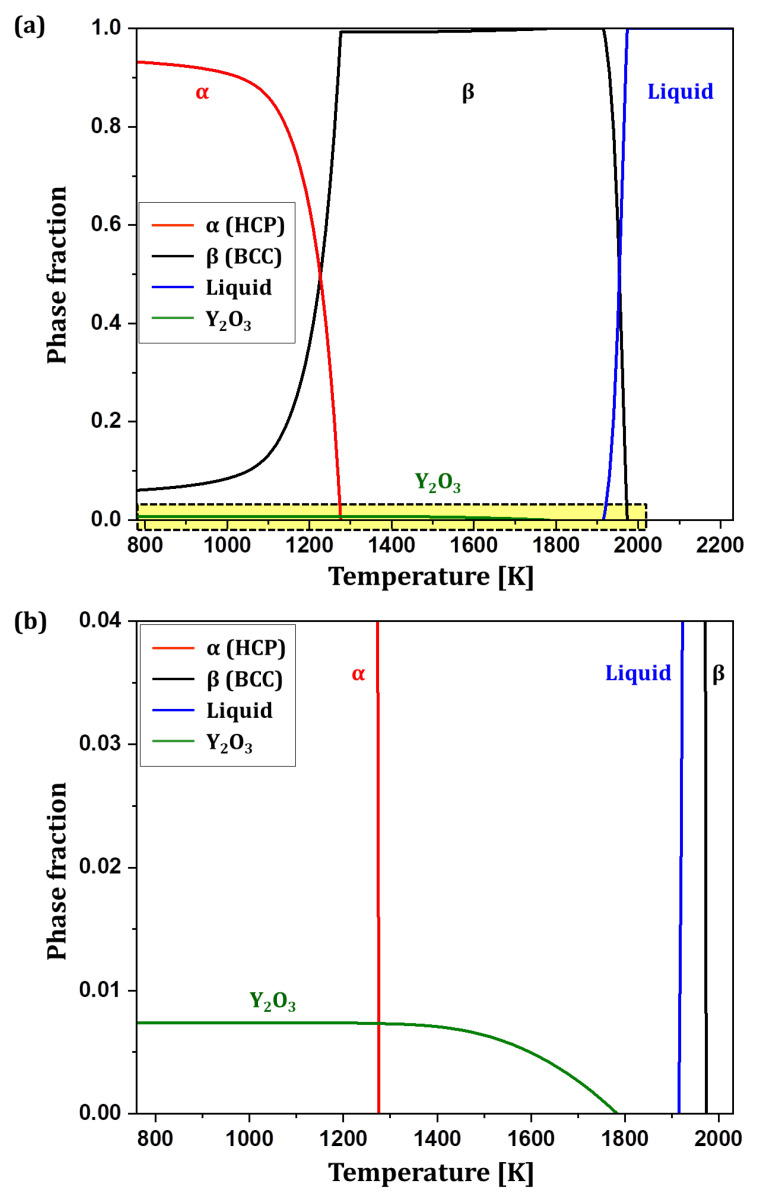
(**a**) Equilibrium phase formation behavior of Ti6242–0.7Y_2_O_3_ and (**b**) its enlarged diagram with the y-axis ranging from 0 to 0.04.

Figure 4a illustrates the equilibrium phase formation behavior of Ti6242–0.7Y_2_O_3_ alloy (0.7 wt% Y_2_O_3_ added to Ti6242 with 0.1 wt% oxygen concentration) as a function of temperature. Red and black lines represent α and β phase fractions, respectively, while the green line represents Y_2_O_3_ phase fraction. Figure 4b provides an enlarged diagram displaying the Y_2_O_3_ phase formation behavior, with the y-axis ranging from 0 to 0.04. The Y_2_O_3_ phase precipitated as a stable phase at low temperatures, with an equilibrium phase fraction of 0.007 (0.7 wt%). The phase fraction began to decrease at temperatures above 1210 K, and the Y_2_O_3_ phase was completely dissolved at 1783 K. The solidus temperature, at which the liquid phase begins to form, was 1915 K. These thermodynamic calculations indicate that Y_2_O_3_ completely dissolves into the Ti6242 molten metal during melting in the gas atomization process. Upon gas spraying to the liquid metal stream for powder fabrication, Y_2_O_3_ will reprecipitate inside the Ti6242 powder, enabling the preparation of oxide-integrated ODS Ti6242 alloy powder.

**Figure 5:**
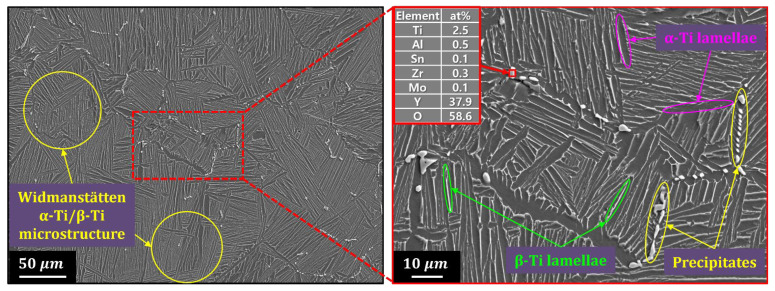
Microstructure of Ti6242–0.7Y_2_O_3_ alloy rod ingot observed by FE–SEM. The left figure is a low-magnification image, and the right figure is a high-magnification image of the red area.

To confirm this possibility, the Ti6242–0.7Y_2_O_3_ alloy powder was fabricated using the in situ gas atomization method. For the powder fabrication by the EIGA, a rod-shaped Ti6242–0.7Y_2_O_3_ alloy ingot was first prepared by VAR, and the ingot’s microstructure is shown in Figure 5. The matrix exhibited a Widmanstätten structure characteristic of Ti alloys [23,26], and white precipitate phases several microns in size were observed along the grain boundaries. The SEM–EDS point analysis of the white precipitate revealed a chemical composition of 2.5Ti-0.5Al-0.1Sn-0.3Zr-0.1Mo-37.9Y-58.6O (at%), very similar to the composition of Y_2_O_3_. This microstructural analysis confirms that Y_2_O_3_ dissolved in molten Ti6242 during melting and coarsely reprecipitated along the grain boundaries during cooling, owing to the relatively slow cooling rate of the VAR process.

**Figure 6:**
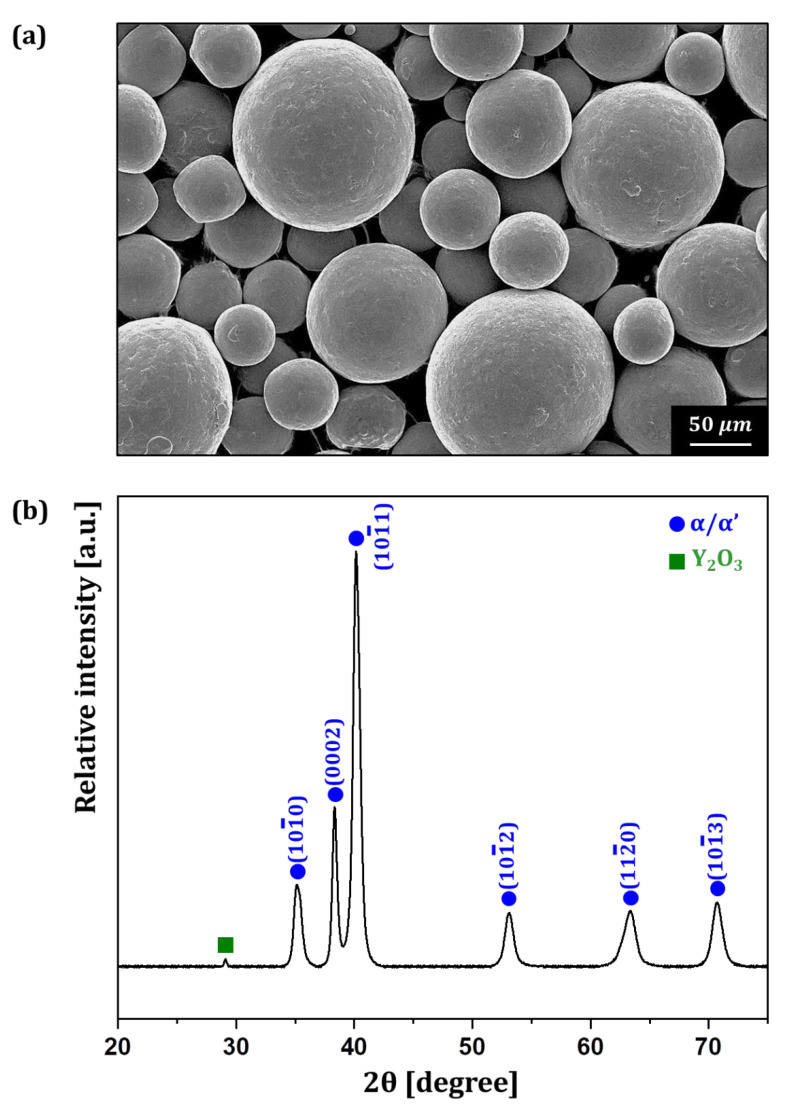
(**a**) The morphology and (**b**) XRD results of the Ti6242–0.7Y_2_O_3_ alloy powder.

The Ti6242–0.7Y_2_O_3_ alloy powder was fabricated via EIGA using the alloy rod ingot. Figure 6a shows the morphology of the Ti6242–0.7Y_2_O_3_ alloy powder observed using the FE–SEM. The powder exhibited a spherical shape and had a smooth surface devoid of visible oxide particles, similar to conventional alloy powders. Conventional spherical Ti alloy powders with a similar powder size exhibit a flowability of 32.16 s/50 g and a packing density of 2.71 g/cm^3^ [27]. The Ti6242–0.7Y_2_O_3_ alloy powder’s flowability and packing density were 30.5 s/50 g and 2.84 g/cm^3^, respectively, values which are similar to those of the conventional Ti alloy powders. Then, to confirm whether Y_2_O_3_ was formed inside the powder, XRD analysis was conducted on the powder, with results shown in Figure 6b. The XRD pattern revealed peaks corresponding to the α phase, while β phase peaks were not detected. In addition, a small intensity peak, observed at a 2θ angle of 29.2°, was identified as corresponding to the Y_2_O_3_.

**Figure 7:**
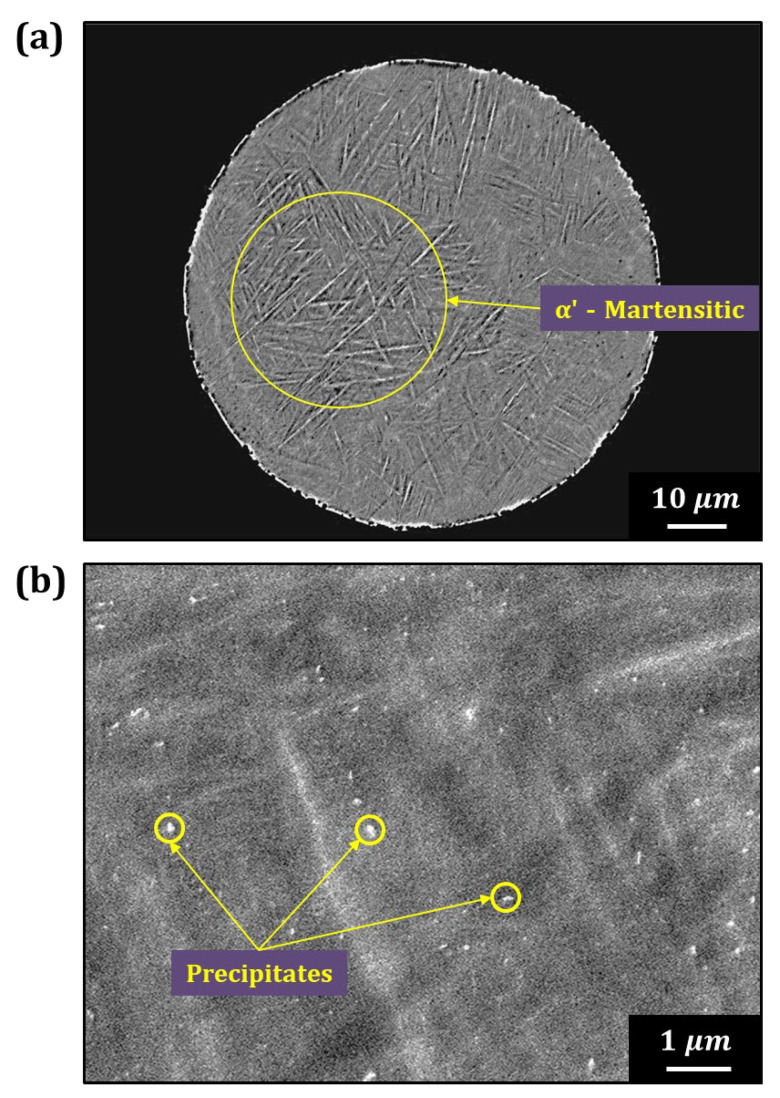
Cross-section microstructure of the (**a**) low magnification and (**b**) high magnification of the Ti6242–0.7Y_2_O_3_ alloy powder observed by the FE–SEM.

Figure 7 displays the cross-section microstructure of Ti6242–0.7Y_2_O_3_ alloy powder using FE–SEM. Figure 7a,b show the low- and high-magnification images, respectively. The low-magnification image (Figure 7a) reveals a needle-like α’ martensitic structure [28,29], formed due to rapid cooling during the EIGA process, with no coarse Y_2_O_3_ phase precipitates observed. The high-magnification image in Figure 7b shows uniformly distributed white precipitates of several tens of nanometers in size. Because the size of the precipitates is too small, SEM–EDS composition analysis was deemed inaccurate due to the interaction volume issues. Therefore, TEM analysis was employed to confirm whether these fine precipitates were Y_2_O_3_ particles.

**Figure 8:**
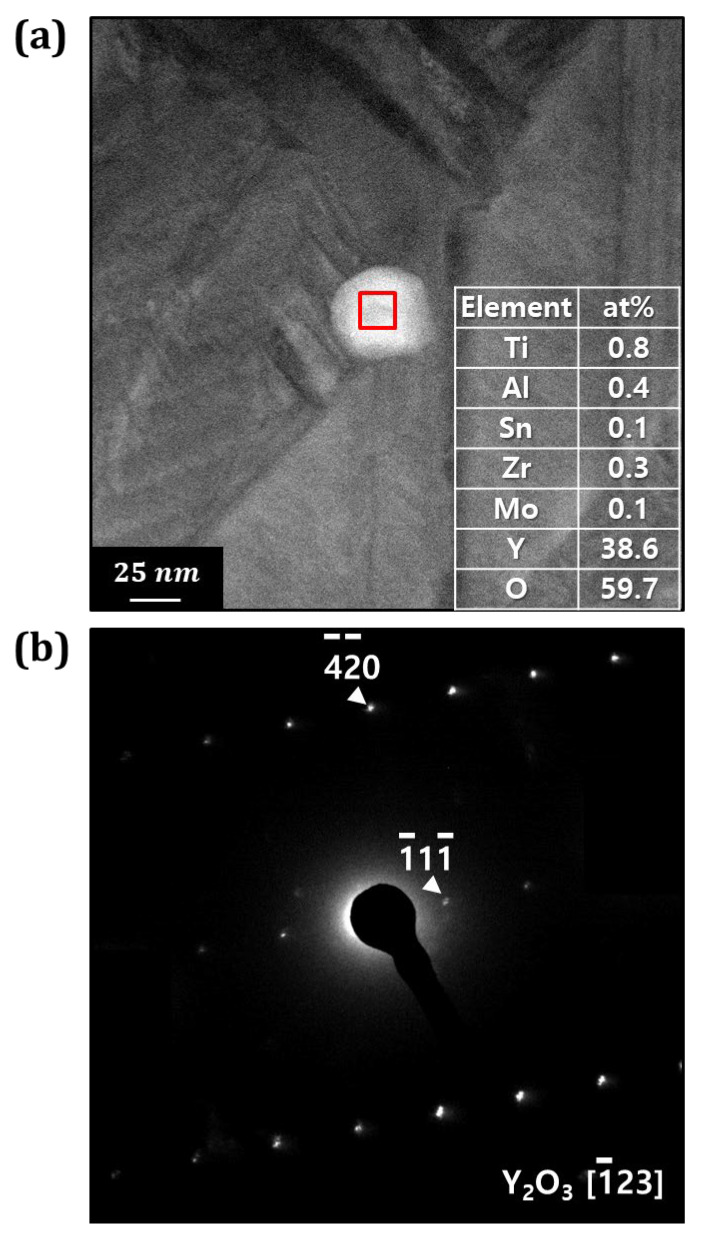
(**a**) Oxide particles inside the Ti6242–0.7Y_2_O_3_ alloy powder observed by FE−TEM and (**b**) its diffraction pattern. The red box in (**a**) indicates the component analysis.

Figure 8a shows an FE–TEM image of the precipitates and their chemical composition as analyzed by TEM–EDS. Precipitates approximately 40 to 50 nm in size were observed within the Ti6242–0.7Y_2_O_3_ alloy powder. Composition analysis confirmed these precipitates as Y_2_O_3_ particles, with a composition of 0.8Ti-0.4Al-0.1Sn-0.3Zr-0.1Mo-38.6Y-59.7O (at%). Figure 8b presents the diffraction pattern of the precipitate phase, further confirming their identification as Y_2_O_3_ particles.

This analysis confirms that fine Y_2_O_3_ particles of several tens of nanometers in size can be uniformly dispersed within the Ti6242 alloy powder using the in situ gas atomization method. Above the melting point of Ti6242–0.7Y_2_O_3_ alloy, the higher oxidation driving force for oxygen dissolution into the Ti6242 matrix causes Y_2_O_3_ to dissolve into the Ti6242 molten metal. During cooling, the driving force reverses, leading to Y_2_O_3_ reprecipitation inside the Ti6242 matrix due to the higher oxidation driving force of Y to form Y_2_O_3_. When preparing the alloy ingot by VAR, Y_2_O_3_ precipitates coarsely along grain boundaries due to the slow cooling rate. In contrast, the rapid cooling induced by high-pressure gas spraying during the gas atomization process results in the uniform formation of fine Y_2_O_3_ particles, several tens of nanometers in size, inside the powder.

**Figure 9:**
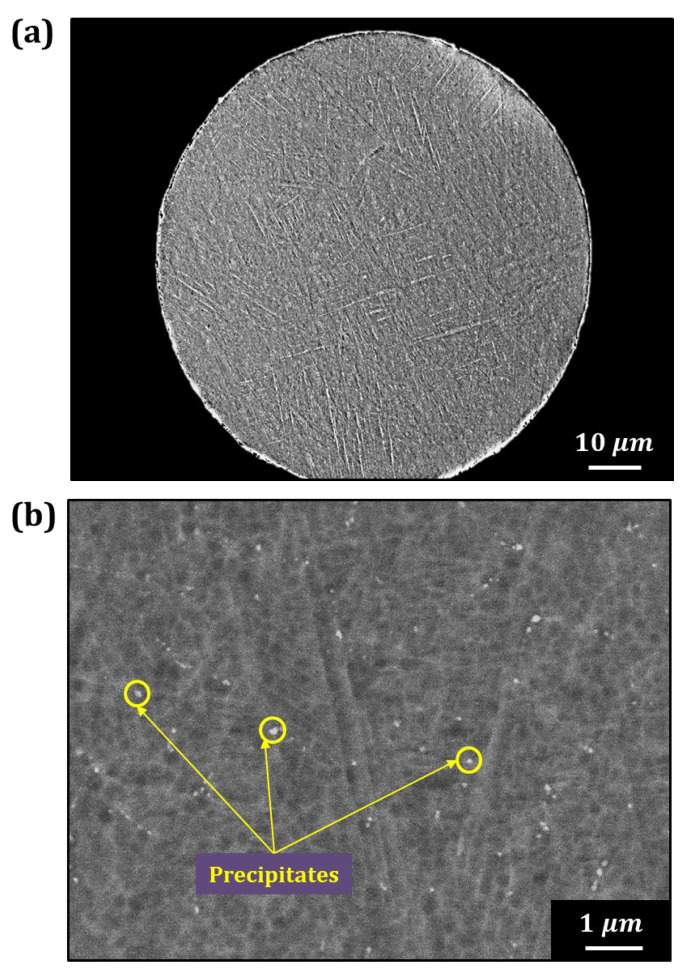
Cross-section microstructure of the (**a**) low magnification and (**b**) high magnification of the Ti6242–0.7Y_2_O_3_ alloy powder after annealing at 600 °C for 10 h.

To examine the thermal stability of the Y_2_O_3_ particles inside the powder, the Ti6242–0.7Y_2_O_3_ alloy powder was annealed at 600 °C for 10 h in a high-vacuum atmosphere. Figure 9a,b show the low- and high-magnification cross-section microstructures of the alloy powder after annealing, respectively. Since the maximum service temperature of the Ti6242 alloy is 538 °C, the alloy powder annealed at 600 °C, slightly higher than the service temperature. Compared to the microstructure of the Ti6242–0.7Y_2_O_3_ alloy powder before annealing, the Y_2_O_3_ particles showed similar size and distribution. According to the thermodynamic calculation results (Figure 4), Y_2_O_3_ particles were stable at low temperature, and they began to dissolve above 1210 K (936.85 °C). Therefore, since the Y_2_O_3_ particles do not dissolve and remain stable up to 936.85 °C, it seems that no coalescence or agglomeration of the Y_2_O_3_ particles occurred even after annealing at 600 °C.

Previous studies explored the use of dispersion materials, such as oxide particles, in additive manufacturing to reduce defects in manufactured parts and enhance mechanical properties through dispersion-strengthening effects. However, these studies primarily utilized powders with dispersion nanoparticles adhered to the powder surface via wet chemical methods or mechanical mixing [19,20]. Such approaches result in several limitations, including the uneven distribution of dispersion materials on the powder surface, reduced powder fluidity, and detachment of dispersion particles during powder flow.

The ODS Ti6242 alloy powder developed in this study addresses these limitations by incorporating fine oxides distributed inside the powder while maintaining a smooth surface comparable to conventional alloy powders used in additive manufacturing. This approach offers several potential advantages, including uniform distribution of dispersion particles throughout the powder and preserved powder fluidity due to the smooth surface. In addition, the ODS Ti6242 powder fabrication technology developed in this study offers a cost advantage, making it suitable for mass production. When manufacturing rod ingots for the EIGA by VAR, the Y_2_O_3_ powder is dissolved during melting and reprecipitated upon cooling, eliminating the need for using expensive nano-sized Y_2_O_3_ powders. Furthermore, there is no increase in process costs, as the powder can be fabricated using the same EIGA process employed for conventional Ti powder production.

## 4. Conclusions

In this study, the fabrication of ODS Ti6242 alloy powder with uniformly distributed fine Y_2_O_3_ particles was achieved using the in situ gas atomization method. The equilibrium phase formation behavior of Ti6242–0.7Y_2_O_3_ alloy was calculated, and the dissolution temperature of Y_2_O_3_ and solidus temperature of the alloy were 1783 and 1915 K, respectively. Ti6242–0.7Y_2_O_3_ alloy demonstrated a thermodynamic relationship wherein Y_2_O_3_ completely dissolved into molten Ti6242 during the melting process of gas atomization and subsequently reprecipitated during the cooling stage of powder production. When fabricating the alloy ingot via VAR, the relatively slow cooling rate resulted in coarse precipitation of Y_2_O_3_ along the grain boundaries. The Ti6242–0.7Y_2_O_3_ alloy powder exhibited a spherical shape with a smooth surface devoid of oxides on the powder surface. The alloy powder exhibited a flowability of 30.5 s/50 g, a value which is similar to that of conventional Ti alloy powders. Cross-sectional analysis of the ODS Ti6242 powder revealed uniform precipitation of fine Y_2_O_3_ particles inside the powder. According to the TEM analysis, the Y_2_O_3_ particles had a size of approximately 40–50 nm. This fine and uniform distribution was attributed to the rapid cooling rate of the gas atomization process.

## Data Availability

The original contributions presented in the study are included in the article, further inquiries can be directed to the corresponding author.
